# Higher-Order Evidence and the Dynamics of Self-Location: An Accuracy-Based Argument for Calibrationism

**DOI:** 10.1007/s10670-022-00589-9

**Published:** 2022-08-27

**Authors:** Brett Topey

**Affiliations:** Department of Philosophy (KGW), University of Salzburg, Salzburg, Austria

## Abstract

The thesis that agents should *calibrate* their beliefs in the face of higher-order evidence—i.e., should adjust their first-order beliefs in response to evidence suggesting that the reasoning underlying those beliefs is faulty—is sometimes thought to be in tension with Bayesian approaches to belief update: in order to obey Bayesian norms, it’s claimed, agents must remain *steadfast* in the face of higher-order evidence. But I argue that this claim is incorrect. In particular, I motivate a minimal constraint on a reasonable treatment of the evolution of self-locating beliefs over time and show that calibrationism is compatible with any generalized Bayesian approach that respects this constraint. I then use this result to argue that remaining steadfast isn’t the response to higher-order evidence that maximizes expected accuracy.

## Introduction

Higher-order defeat—i.e., defeat by evidence suggesting that some of one’s own beliefs have been produced by faulty reasoning—is sometimes thought to be a source of tension with Bayesian approaches to belief update. Consider, for instance, a version of the classic case of the pilot who discovers she’s hypoxic:

Hypoxia Aisha is flying a plane at 10 am on Monday, and the evidence she’s gained since 8 pm on Sunday, before her flight, is $E \wedge D$. *E* is evidence of the usual kind, and Aisha, on doing some calculations based on *E*, reaches the verdict that *H* is true, where *H* is the proposition that she has enough fuel to reach an airstrip much farther away than her original destination. In the usual circumstances, this verdict would induce her to become highly confident in *H*. But these aren’t the usual circumstances: Aisha also has the evidence *D*, which, though it doesn’t bear directly on *H* in any way, does indicate that she’s suffering from hypoxia, a condition that occurs at high altitudes and diminishes pilots’ ability to reason, causing them to commit random errors. (We can suppose, for concreteness, that when pilots suffering from hypoxia do calculations like the one Aisha has just done, they reach the correct verdicts only half the time.) As it happens though, Aisha, in this case, has completed the calculations correctly.^1^

According to an exceedingly plausible view known as *calibrationism*, rationality requires that Aisha’s confidence in *H*, on gaining *E* along with the higher-order evidence *D*, be significantly lower than it would have been had she gained only *E*—to fail to calibrate her beliefs in this way would be to continue to insist on the correctness of her calculations despite her knowledge that they’re hypoxia-affected, and this sort of insistence, it seems, would amount to nothing more than pigheaded refusal to acknowledge the dangerous nature of her situation. On the face of it, though, calibrating in this way would require violating conditionalization.^2^ Let the probability function $p_S$ model Aisha’s belief state at 8 pm on Sunday. Then $p_S(H \mid E)$ is extremely high. Furthermore, it should be the case that $p_S(H \mid E \wedge D) = p_S(H \mid E)$; the supposition that Aisha will get evidence indicating she’s hypoxic on Monday should make no difference to her confidence in her calculations on Sunday, when she *knows* she’s not hypoxic. $p_S(H \mid E \wedge D)$, then, should be extremely high as well. (Note that she need not actually do any calculations on Sunday—her conditional credences may simply be, for example, abstractions from her dispositions. Indeed, if she *does* do the relevant calculations on Sunday, we must stipulate, in order for the case to have its intended force, that one of the effects of hypoxia is to distort one’s memories of previous calculations of this sort in such a way that they are no longer to be trusted. Otherwise, she could remain confident in *H* despite gaining *D* just by relying on her memory of the calculations she did on Sunday, without needing to rely on any hypoxia-affected calculations.) So, if rationality requires that she calibrate—i.e., if $p_M(H)$ should *not* be extremely high, where $p_M$ models her belief state at 10 am on Monday, after she has gained $E \wedge D$—then $p_M(H)$ should be less than $p_S(H \mid E \wedge D)$, which means rationality requires that she violate conditionalization. Our exceedingly plausible calibrationist view, then, appears to be straightforwardly incompatible with Bayesianism.

It gets worse. We know that, insofar as what Aisha cares about is accuracy, she should take conditionalizing to be the optimal response to new evidence—this is an immediate consequence of Greaves and Wallace’s (2006) proof that conditionalization uniquely maximizes expected accuracy. (We assume here, as usual, that Aisha assigns accuracy scores in a *strictly proper* way—i.e., in such a way that every probabilistically coherent credence function turns out to be self-recommending, in the sense that it expects itself to be more accurate than alternative credence functions.) So, insofar as she should calibrate (and so should violate conditionalization), rationality requires that she *not* maximize expected accuracy. It appears then, that calibrationism, in virtue of its incompatibility with Bayesianism, is also incompatible with the accuracy-first program in epistemology.

What’s the lesson here? We might suppose that we should simply abandon calibrationism in favor of the view that, whatever higher-order evidence Aisha gains, the rational thing for her to do is to remain *steadfast* in her beliefs.^3^ But this would be hasty—there’s reason to suspect that, despite appearances, Bayesianism and calibrationism can be reconciled. The thought is that the incompatibility here is merely apparent, an artifact of our failure to take adequate account of a category of belief that, as Christensen (2010a) points out, plays a crucial role in cases of higher-order defeat: self-locating belief. When Aisha gains *D* on Monday, she gains evidence suggesting that her *present* belief state is the product of hypoxia-affected reasoning—*D* is evidence about her reasoning capacities at 10 am on Monday and so tells her something about the situation she’s in *now*. But this is only because she knows that it’s *now* 10 am on Monday: when she’s sitting at home at 8 pm on Sunday (and knows that she is), the supposition that she’ll gain *D* when she’s flying on Monday, even if it’s true, suggests nothing at all about her present belief state. The mismatch between what $p_S(H \mid E \wedge D)$ should be and what $p_M(H)$ should be, then, is closely connected to the fact that Aisha’s self-locating beliefs evolve between Sunday and Monday. Furthermore, it’s well known that self-locating beliefs make trouble for the conditionalization principle, which wasn’t designed with such beliefs in mind. For instance, to lose certainty is to violate conditionalization (as standardly formulated), but an agent might, without irrationality, lose certainty in the belief that it’s 12:30, just because some time has passed.^4^ It would be natural, then, to suspect that “when we fully understand how belief updating works in contexts where self-locating beliefs are important, we will see that [higher-order evidence]-involving cases can be accommodated in a formal account of belief updating which preserves a general matching between present credences on the supposition that E, and future credences on learning just E” (Christensen, 2010a, p. 201)—i.e., that a Bayesian approach, appropriately generalized so as to provide a reasonable framework for handling the evolution of self-locating beliefs, will prove to be compatible with calibrationism after all.

I argue here that this suspicion is correct. In particular, I introduce and motivate a minimal constraint on a reasonable treatment of the dynamics of self-locating belief, and then I show that any Bayesian approach that can meet this constraint will thereby be compatible with calibrationism.

Even if this is correct, though, there’s an additional worry. Though Greaves and Wallace have shown that conditionalization is the update procedure that maximizes expected accuracy, their result doesn’t straightforwardly apply in cases where self-locating beliefs are important: they rely on a framework in which the objects of credence are propositions understood as sets of possible worlds, and such propositions can’t encode self-locating information. So even if a Bayesian approach, appropriately generalized to handle self-location, is compatible with calibrationism, there remains the question of whether updating in accordance with this approach’s recommendations will turn out to maximize expected accuracy. And Schoenfield (2018) suggests that it won’t: she argues that what maximizes accuracy, when self-locating beliefs are in play, isn’t conditionalization but an alternative procedure, one that’s itself incompatible with calibrationism. If this is right, then we must either give up calibrationism or abandon the accuracy-first program in epistemology.^5^

As we’ll see, though, this further worry is answerable: Schoenfield’s argument notwithstanding, updating in accordance with the recommendations of a suitably generalized Bayesian approach *does* maximize expected accuracy. In fact, when we take proper account of the dynamics of self-location, it becomes clear that the accuracy-first program, far from being incompatible with calibrationism, *motivates* that view. So what we have, in the end, is an accuracy-based argument for calibrationism along with an assurance that endorsing this view doesn’t require us to abandon a Bayesian approach to belief update.

The paper is organized as follows. We begin in Sect. 2 with a critical discussion of Schoenfield’s argument that calibration fails to maximize expected accuracy. Then, based on this discussion, Sect. 3 introduces and motivates a minimal constraint on an adequate treatment of the dynamics of self-location. Section 4 explains why any generalized Bayesian approach that can meet this constraint will thereby be compatible with calibrationism, and Sects. 5–6 provide, on this basis, an accuracy-based argument for calibrating. Finally, we conclude in Sect. 7 with a brief discussion of a surprising implication of the picture at which we’ve arrived, an implication that bears on another debate about the dynamics of self-location.

## Schoenfield’s Argument

On Schoenfield’s gloss, Christensen’s claim about the importance of self-locating beliefs in cases of higher-order defeat amounts, when applied to Aisha’s case, to the claim that Aisha’s higher-order evidence is not to be understood as the ordinary proposition *D* but as a centered proposition $D_c$, a self-locating proposition Aisha would express using some sentence like “I’m hypoxic now” (2018, p. 698). The resulting explanation of why Aisha can calibrate without violating conditionalization is simple: $D_c$, as evaluated by Aisha at 8 pm on Sunday, is true just in case she’s hypoxic *at 8* *pm* *on Sunday*. (A centered proposition is here understood as a set, not of possible worlds, but of centered worlds, where a centered world is a triple consisting of a possible world, an agent, and a time.) So at 8 pm on Sunday, she should believe, on supposing $D_c$, that her present belief state is indeed the product of hypoxia-affected reasoning. We can plausibly claim, then, that $p_S(H \mid E \wedge D_c)$ should be less than $p_S(H \mid E)$, in which case calibrating doesn’t require violating conditionalization after all.

The problem with this explanation, says Schoenfield, is that, insofar as the evidence Aisha gains between 8 pm on Sunday and 10 am on Monday is indeed $E \wedge D_c$, conditionalizing on that evidence fails to maximize expected accuracy. Greaves and Wallace’s proof that conditionalization is maximizes expected accuracy might be thought to show otherwise, but that proof, Schoenfield observes, relies on a particular assumption about what agents know about their future evidence and so isn’t obviously applicable here. Where *P* is an evidence proposition an agent might gain on undergoing a learning experience over a particular period of time, let *L*(*P*) be the proposition that the evidence the agent gains, on undergoing the learning experience in question, is *P*. Then Greaves and Wallace’s assumption is that the agent satisfies the following condition:

Evidential Completeness Before undergoing the learning experience in question, the agent is certain that, for any evidence *P* she might gain, $P \leftrightarrow L(P)$.^6^

In other words, conditionalization maximizes expected accuracy only on the assumption that the agent is certain that, for any evidence proposition *P* she might gain on undergoing a learning experience over a particular period of time, she’ll gain *P* just in case it’s true. (This assumption derives its plausibility from the thought that an ideal agent can be sure that the truth values of evidence propositions will be transparent to her, perhaps because evidence propositions will always be propositions about her own experience. It seems that all parties to the discussion here are willing to accept this thought, at least for the sake of argument.) Without this assumption, Schoenfield explains, what can be shown is that the procedure that maximizes expected accuracy is a procedure she calls conditionalization*, which amounts to conditionalizing not on *P* itself but on *L*(*P*).^7^ That is, in the general case, the agent, in order to maximize expected accuracy, should conditionalize not on the evidence proposition *P* but on the proposition *that the evidence she gains during the learning experience is P*. (In cases where Evidential Completeness is satisfied, the agent takes *P* and *L*(*P*) to be equivalent, which means conditionalization and conditionalization* come to the same thing. This is why conditionalization maximizes expected accuracy in these cases.)

This doesn’t yet show that conditionalization fails to maximize expected accuracy in cases of higher-order defeat—conditionalization fails to maximize expected accuracy only if the agent doesn’t satisfy Evidential Completeness and so doesn’t regard *P* and *L*(*P*) as equivalent. Schoenfield’s final step here, then, is to argue that agents like Aisha do indeed fail to satisfy Evidential Completeness.

This part of the argument begins with the observation that what Evidential Completeness requires, in Aisha’s case, is that, before undergoing the learning experience she undergoes between 8 pm on Sunday and 10 am on Monday, she take $L(E \wedge D_c)$ to be equivalent to $E \wedge D_c$. But $L(E \wedge D_c)$ is just the proposition that the evidence Aisha gains, on undergoing the learning experience she undergoes between 8 pm on Sunday and 10 am on Monday, is $E \wedge D_c$, where this latter proposition is a centered proposition she’d express, *at 10* *am* *on Monday*, as “*E* and I’m hypoxic now”. Let’s assume—as, again, all parties here are willing to do—that Aisha is certain that the truth values of evidence propositions are transparent. Because centered evidence propositions are in play, this assumption, notice, doesn’t entail that Aisha satisfies Evidential Completeness: for her to satisfy Evidential Completeness is for her to be certain that, for any evidence proposition she might gain, she’ll gain it just in case it *is* true relative to *her now*, but for her to be certain that the truth values of evidence propositions are transparent is for her to be certain that, for any evidence proposition she might gain, she’ll gain it just in case it *will be* true relative to her *when she gains it*.^8^ So, given our assumption, what Aisha is certain of, before undergoing the learning experience, is that $L(E \wedge D_c)$ is true just in case *E* and Aisha is hypoxic *at 10* *am* *on Monday*. That is, at 8 pm on Sunday, she takes $L(E \wedge D_c)$ to be equivalent to $E \wedge D$, where *D* is, again, the non–self-locating proposition that Aisha is hypoxic at 10 am on Monday. ($D_c$, again, is true relative to a person and a time, but the source of the problem here is the temporal relativity. Aisha, we can suppose, remains certain at all times that *she* is Aisha.)

This tells us two things. First: in order to satisfy Evidential Completeness, Aisha, before undergoing the learning experience, must take $D_c$ to be equivalent to *D*. Or, in other words, she must be certain, at 8 pm on Sunday, that $D_c \leftrightarrow D$, where this is a proposition she’d express as “I’m hypoxic now just in case Aisha is hypoxic at 10 am on Monday”. But she surely isn’t certain of *that*—the fact that she isn’t hypoxic while sitting in her house on Sunday night just doesn’t have any bearing at all on whether she’ll be hypoxic during her flight on Monday. So she doesn’t satisfy Evidential Completeness. Second: since Aisha is certain, at 8 pm on Sunday, that $E \wedge D \leftrightarrow L(E \wedge D_c)$, conditionalizing on $L(E \wedge D_c)$ amounts to conditionalizing on $E \wedge D$. So it appears that adopting a picture on which higher-order evidence propositions are self-locating can’t help us at all—agents, in order to maximize expected accuracy, must conditionalize on non–self-locating analogs of those propositions anyway.

Here’s a more intuitive way of seeing the point here. When Aisha supposes, at 8 pm on Sunday, that $L(E \wedge D_c)$, what she’s supposing is something that, as she knows, is true just in case *E* and *Aisha* is hypoxic *at 10* *am* *on Monday*. (Again, we’re assuming that she knows that the truth values of evidence propositions are transparent.) But as we’ve already discussed, even if it’s true that Aisha will be hypoxic at 10 am on Monday, this fact suggests nothing about her ability to calculate at 8 pm on Sunday, when she knows she’s not hypoxic. So $p_S(H \mid L(E \wedge D_c))$ should be equal to $p_S(H \mid L(E))$. And since conditionalization* is the procedure that maximizes expected accuracy, Aisha, in order to maximize expected accuracy, should remain steadfast.

Now: there’s a great deal of material here that merits discussion, and we’ll return to some of the details in Sect. 5 below. For the moment, though, what I want to point out is this: Schoenfield, in setting up her argument, treats Christensen’s claim that self-locating belief is important in cases of higher-order defeat as if it’s equivalent to the claim that higher-order evidence is itself to be understood as self-locating, and so she takes her target to be a view on which Aisha ought to conditionalize on $E \wedge D_c$—i.e., a view on which $p_M(\cdot )$ should be equal to $p_S(\cdot \mid E \wedge D_c)$. The reason this is significant is that there are conclusive reasons for thinking this interpretation can’t be quite right—the view here can’t really be what Christensen has in mind, for the simple reason that, even independently of accuracy considerations, it’s very obviously incompatible with any plausible picture of the dynamics of self-location.

To see why, consider that nothing in the setup of Aisha’s case suggests that Aisha is ever less than sure where she is in time. We can suppose, then, that she perfectly tracks time, down to the moment: on Monday, at the moment when she updates on her evidence, she’s certain that it’s now 10 am on Monday. So, since she’s also (we’re supposing) certain that she’s Aisha, she is, at this moment, certain that she’s hypoxic now just in case Aisha is hypoxic at 10 am on Monday—i.e., that $D_c \leftrightarrow D$. But if that’s right, the view Schoenfield takes to be her target fails for trivial reasons: insofar as Aisha, at the moment when she updates on her evidence, takes $D_c$ and *D* to be equivalent, there’s no difference, from her perspective, between gaining $E \wedge D_c$ and gaining $E \wedge D$, which means that insisting on a picture on which she gains $E \wedge D_c$ rather than $E \wedge D$ is certainly not going to be of any help to us. Something seems to have gone badly wrong.^9^

What, exactly, is going on here? Returning to Christensen’s discussion will help us to diagnose the problem. The particular case he’s discussing isn’t Aisha’s case—it’s a structurally analogous case in which a scientist, on going into the lab on Monday, gains the evidence $E \wedge D$, where *E* is first-order evidence bearing on a hypothesis *H* and *D* is the higher-order evidence that a reason-distorting drug was slipped into the scientist’s Monday breakfast. What Christensen says about the case is this:

The way my beliefs should evolve depends crucially on my knowing my temporal location. If we take D as “I’m drugged Monday at breakfast,” then D will undermine my confidence in H when I get to the lab only because I’ll be confident that it’s Monday morning. But on Sunday, I’m obviously not confident of *that*. (2010a, p. 201)

In other words, the scientist, on gaining the higher-order evidence *D*, should become confident that he’s presently impaired, but the explanation isn’t that *D* is self-locating. It’s instead that his *background knowledge*—in particular, his knowledge of temporally self-locating information, information about where he is in time—evolves between Sunday and Monday in such a way that the very same higher-order information, though it suggests nothing about his present belief state when he considers it on Sunday night, does suggest something about his present belief state when he considers it on Monday morning.

The thought here, then, seems to be not that higher-order evidence is itself self-locating but that the evolution of *other* self-locating beliefs plays an important role in the phenomenon of higher-order defeat. And if that’s right, Schoenfield’s argument fails to refute the picture Christensen actually sketches, for the simple reason that the argument doesn’t target that picture at all. So that picture still stands in need of evaluation. But before we can evaluate it, we need to understand a bit more about the dynamics of the relevant self-locating beliefs.

## The Dynamics of Self-Location

As we saw in considering Aisha’s case, higher-order defeat doesn’t require that agents be susceptible to losing track of time, nor does it require that they be susceptible to becoming less than certain about who they are. So, for our purposes, there’s no need to discuss in any detail how to model agents who have these susceptibilities. (Indeed, modeling such agents is going to present its own problems, problems that have nothing to do with higher-order evidence, and we would do well to disentangle these problems from the question of how to model cases of higher-order defeat.) So we’re going to focus on agents who, first, never lose track of time and, second, always remain certain of who they are. We’ll also assume for simplicity that, third, the agents in question have some canonical way of referring to locations in time. Call agents who meet these criteria *self-certain*. The rest of our discussion will proceed under the simplifying assumption that Aisha is a self-certain agent.

Our question, then, is how to model the evolution of the beliefs of self-certain agents over time. Up to now we’ve been assuming, as is usual in the recent epistemological literature, that self-locating beliefs are to be understood as attitudes toward centered propositions. And on this way of understanding self-locating beliefs, it’s trivially easy to model a self-certain agent’s continued certainty about her own identity. Aisha’s certainty that she’s Aisha, for instance, can be modeled as a maximal credence, maintained over time, in the centered proposition containing every world the agential center of which is Aisha.

Modeling an agent’s temporally self-locating knowledge, though, is much more difficult. The problem is that the objects of temporally self-locating belief are, on this framework, centered propositions that change in truth value over time, which means that to maintain the same attitudes toward these propositions as time passes is to fail to keep track of time—successful timekeeping requires that agents engage in continuous revision of a wide variety of their beliefs. If a self-certain agent is at one moment certain that she’s at time *t*, this certainty can be modeled as a maximal credence in the centered proposition containing every world the temporal center of which is *t*. But if some amount of time *n* passes, she’ll no longer have a maximal credence in that proposition. She’ll instead have a maximal credence in a different centered proposition, one containing every world the temporal center of which is $t+n$. And similarly for her other temporally self-locating beliefs. Suppose, for instance, that at *t* she has a particular credence in the centered proposition she’d express by “The cat is on the mat now”—i.e., the proposition containing every world in which, at the time on which that world is centered, the cat is on the mat—and suppose that, between *t* and $t+n$, she doesn’t gain any new evidence relevant to whether the cat was on the mat at *t*. Then she should, at $t+n$, have that same credence in the different centered proposition she’d express by “The cat was on the mat *n* ago”, where this proposition contains every world in which, *n* before the time on which that world is centered, the cat was on the mat.

More generally, if an agent has a temporally self-locating attitude at time *t*, its object, on this framework, is a centered proposition *P*. But the associated attitude at $t+n$ (where *n* may be positive or negative) is not her attitude toward *P*—it’s an attitude toward a related centered proposition $P^n$, where $P^n$ is true at a centered world $\langle w, a, t+n \rangle$ just in case *P* is true at $\langle w, a, t \rangle$. We’ll call this related proposition the *n*-*shifted counterpart* of *P*. (Note that, for any attitude that’s *not* temporally self-locating, the proposition that is its object will be such that the time on which a world is centered makes no difference to the proposition’s truth value. So, for any such proposition, $P = P^n$.)

The framework here, in short, is one on which failure to keep track of time is the default condition—an agent who can successfully keep track of time is thereby an agent a wide variety of whose beliefs are in a constant state of flux. As a result, the dynamics of temporal self-location are apt to seem mysterious, especially if we’re sympathetic to the evidentialist assumption that rational agents revise their beliefs only in response to new evidence: on that assumption, a rational agent, in order to keep track of time, must be responsive not only to the usual perceptual evidence about what the world is like but also to an additional continuous stream of temporal evidence that needs to be processed, not by conditionalization as standardly formulated, but in some different way. (Again, conditional credences, as standardly understood, preserve certainty, but we’ve seen that keeping track of time involves losing certainty in, for instance, the centered proposition an agent would express by “It’s now time *t*”.)

This difficulty aside, though, it’s clear enough that the continuous revision required here is highly systematic—even on a framework that emphasizes flux, we can see that keeping track of time involves a kind of stability as well. And as it turns out, there’s a way of modeling self-location that brings this stability to the fore: rather than understanding self-locating beliefs as attitudes toward centered propositions, we might understand them as attitudes toward *Fregean thoughts*. In particular, we might understand *temporally* self-locating beliefs as attitudes toward *dynamic* Fregean thoughts of the sort described by Gareth Evans (1981): thoughts an agent’s grasp of which depends on her ability to keep track of time.

In general, a Fregean thought is composed of the senses of the words one would use to express that thought, where the sense of an expression (in a context) is a particular mode of presentation, or way of thinking, of that to which the expression (in that context) refers. And if self-locating beliefs are understood as attitudes toward thoughts of this sort, modeling an agent’s certainty about her own identity is, as before, trivially easy. When an agent grasps a first-personal thought, she is thinking of herself in a particular way: from the inside, so to speak.^10^ So we can model Aisha’s certainty that she’s Aisha, for instance, as a maximal credence, maintained over time, in the first-personal Fregean thought she’d express by “I am Aisha”.

As for temporally self-locating knowledge: note first that the dynamic thoughts described by Evans are thoughts about a particular moment in time such that the agent’s way of thinking about the moment, the mode in which it’s presented to her, is a product of her ability to keep track of time. The idea is that, when the agent thinks of some moment as *now* and then one minute later thinks of it as *one minute ago*, she’s thinking of that moment in the same way—roughly, the keeping-track-of-time way—both times. The framework here, as Evans explains, is one on which

being in the same epistemic state may require different things of us at different times; the changing circumstances force us to change in order to keep hold of a constant reference and a constant thought—we must run to keep still. From this point of view, the acceptance on $d_2$ of ‘Yesterday was fine’, given an acceptance on $d_1$ of ‘Today is fine’, can manifest the *persistence* of a belief in just the way in which acceptance of different utterances of the same sentence ‘The sun sets in the West’ can. (1981, p. 293)

That is, when an agent on one day has a belief that she’d express by “Today is fine” and then, by virtue of successfully keeping track of time, has a belief on the next day that she’d express by “Yesterday was fine”, we can understand her as being related in slightly different ways to the very same Fregean thought, and so, insofar as her credence in that thought doesn’t change between the first day and the second, we can understand her as having a single belief that persists over time rather than as having a succession of beliefs that are systematically related despite having different objects.^11^

On this alternative framework, successful timekeeping is primarily a matter of belief maintenance rather than of belief revision—since dynamic Fregean thoughts don’t change in truth value over time, agents’ beliefs aren’t required to be in a constant state of flux. So the dynamics here turn out to be relatively simple. For instance, since the agent in the above case of the cat and the mat is successfully keeping track of time, the dynamic Fregean thought expressed at *t* by “The cat is on the mat now” is the very same thought as that expressed at $t+n$ by “The cat was on the mat *n* ago”, and so, unless the agent gains information that’s relevant to whether the cat was on the mat at *t*, her credence in this thought shouldn’t change at all—she should simply maintain her belief.

This, then, is a framework on which keeping track of time is the default condition—there’s no need to suppose that successful timekeeping involves continuous updating of a wide variety of beliefs in response to a stream of mysterious temporal evidence. We can suppose instead that no special story of what successful timekeeping involves is required at all; if there’s anything mysterious or difficult to model about temporal self-location, it’s the phenomenon of *losing* track of time, not the phenomenon of *keeping* track of time.^12^

Of course, strictly speaking, a self-certain agent’s credences in Fregean thoughts can’t on their own model her belief state at a given time—her certainty about where she is in time isn’t representable as an attitude toward a dynamic Fregean thought. The problem is that the dynamic thought that she’d express at *t* by “It’s now time *t*” is the very same thought that she’d express at $t+n$ by “Time *t* was *n* ago” and at $t-n$ by “Time *t* will be in *n*”; this thought doesn’t change in truth value as time passes. So her credence in this thought should remain the same over time, which means this credence can’t be used to represent her knowledge of her temporal location as that location changes. In order to fully represent her belief state, then, we must include, in addition to her credences in Fregean thoughts, some representation of her knowledge of what moment is the present moment. Let us, then, model her belief state, not just as a probability function, but as an ordered pair $\langle p, t \rangle$, where *p* is a probability function and *t* denotes the time she knows to be the present time.^13^

Something similar goes for modeling suppositional beliefs. An agent can, after all, engage in suppositional reasoning not only about evidence she might gain about the world but also about the passage of time—she can, so to speak, suppositionally project herself into the future—and so we need to be able to model the beliefs she has conditional on the supposition that some amount of time has passed. On the usual centered-proposition framework, we plausibly can model these beliefs as beliefs conditional on the supposition that the only new evidence she’s gained is a stream of temporal evidence, where this amounts to taking any belief she has at time *t* on supposing that *n* has passed to be a belief conditional on the supposition that, for every centered proposition in her total body of evidence at *t*, its *n*-shifted counterpart is true instead. (Admittedly, these suppositional beliefs can’t be modeled as conditional probabilities of the usual sort, since the agent is supposing true something she’s certain is false. The point, for now, is just that there’s going to be *some* way to represent an agent’s projection of herself into the future in terms of her attitudes toward centered propositions.) But there’s no analogous way to model these beliefs as beliefs conditional on the supposition that some Fregean thought is true, since, again, dynamic Fregean thoughts don’t change in truth value as time passes. Some other way of representing agents’ projection of themselves into the future is needed. Let us, then, redefine our probability function so that it isn’t a binary function that returns a probability given some proposition *P* and some (possibly empty) evidence proposition *E* but is instead a ternary function $p(P \mid E, n)$, where *n* is a time adjustment factor representing how far in the future the agent is supposing herself to be. (We can then understand ‘*p*(*P*)’ and ‘$p(P \mid E)$’ as abbreviations of ‘$p(P \mid \top , 0)$’ and ‘$p(P \mid E, 0)$’, respectively.)

I don’t mean to suggest, in introducing this Fregean framework, that it’s correct and the centered-proposition framework incorrect, nor do I mean to suggest that the Fregean framework is better suited for every purpose than the centered-proposition framework is. The point is just that a second way of modeling the self-locating beliefs of self-certain agents is indeed available—these agents’ temporally self-locating beliefs exhibit a kind of stability that isn’t entirely obvious on the centered-proposition framework, and the Fregean framework allows us to make this stability explicit, if we’re interested in doing so. The reason this is significant for our purposes is that making this stability explicit allows us to parse out changes in belief that occur in response to the passage of time from those that occur in response to new evidence about the world, and treating these changes separately makes it relatively easy to see that there are certain restrictions on what results a reasonable belief update procedure may deliver in cases of self-certainty.

Note that, if an agent is self-certain, her self-locating beliefs, if we model them as attitudes toward Fregean thoughts, are, from the agent’s own perspective, equivalent to beliefs in standard non–self-locating propositions. If the agent is certain that she’s Sheila and that it’s 3 pm on October 1, 1989, then the content of the belief she’d express as “I’m hungry now”, if we model that content as a dynamic Fregean thought, is equivalent, from her perspective, to the non–self-locating proposition that Sheila is hungry at 3 pm on October 1, 1989—she can be certain, no matter how much time passes, that the belief in question is true if and only if Sheila was hungry at 3 pm on October 1, 1989. And the same goes for any evidence she might gain—even if the evidence is temporally self-locating, we can model it as a dynamic Fregean thought, in which case it’s going to be equivalent, from the agent’s own perspective, to a standard non–self-locating evidence proposition.

In short, since cases of self-certainty are cases in which the agent is always sure who she is and where she is in time, they’re cases in which the Fregean framework allows us, in considering how she should update on new evidence, to set self-locating information aside completely: any new evidence she gains is going to be best understood as new evidence about what the world is like, and updating on that new evidence is going to be best understood as updating her beliefs about what the world is like. And we know already that the right way to update one’s beliefs about what the world is like in the face of new information about what the world is like—i.e., the right way to update when self-locating information is *not* in play—is by conditionalization. So, if we model the objects of belief as Fregean thoughts, we can conclude that, if the agent, on undergoing a learning experience between *t* and $t+n$, gains evidence modeled by the Fregean thought $E_F$ and then updates on that evidence at $t+n$, then it should be the case that $p_{t+n}(\cdot ) = p_{(t+n)-}(\cdot \mid E_F, 0)$, where $\langle p_{(t+n)-}, t+n \rangle$ models her state just before she updates and $\langle p_{t+n}, t+n \rangle$ models her state just after.^14^

Of course, what we’re really interested in is how her state just after she updates on $E_F$ should relate to her state at *t*, before she undergoes the learning experience. So there remains the question of how her attitudes should evolve between *t* and $t+n$, *before* she updates on $E_F$—i.e., of how her state at *t* should relate to the state she’s in just before she updates. And this isn’t a trivial question to answer, especially since we haven’t yet said much of substance about what, in general, it takes to respond rationally to the passage of time. But consider: given how we’ve characterized suppositional projection of oneself into the future, there aren’t any formal constraints on what the relationship should be between an agent’s unconditional credences and her credences conditional on this sort of projection. So we can simply stipulate that our agent is disposed to respond rationally to the passage of time, whatever responding rationally to the passage of time amounts to, and that these dispositions are encoded into her future-projected credences. And if we do so, our answer here becomes very simple: the attitudes our agent has just before she updates should be the same as the attitudes she has at *t* conditional on the supposition that *n* has passed. So it should be the case that $p_{(t+n)-}(\cdot \mid E_F, 0) = p_{t}(\cdot \mid E_F, n)$, where $\langle p_t, t \rangle$ models her state at *t*.^15^ And that means it should be the case that $p_{t+n}(\cdot ) = p_{t}(\cdot \mid E_F, n)$. That is, the attitudes toward Fregean thoughts she has just after updating should match the future-projected conditional-on-$E_F$ attitudes she has at *t*.

Once we’ve used the Fregean framework to arrive at this result, it’s relatively straightforward to translate it back into the more familiar language of the centered-proposition framework. Suppose again that our self-certain agent is in the situation described above: she gains some evidence between *t* and $t+n$ and then updates on that evidence at $t+n$. Then the question is how the agent’s attitude toward a given proposition *P* at $t+n$, just after she updates on an evidence proposition *E*, should be related to her attitudes at *t*, where all of her attitudes are understood as attitudes toward centered propositions.

The key thing to keep in mind here is that, though we’re working in the centered-proposition framework, we already know how to model all of the agent’s attitudes according to the Fregean framework as well. So, in particular, the credence she has in *P* just after updating can equally well be expressed as a credence in a Fregean thought $P_F$. And that means it should be the case that $p_{t+n}(P) = p_{t+n}(P_F)$. Furthermore, we’ve already seen that $p_{t+n}(P_F)$ should be equal to $p_t(P_F \mid E_F, n)$, where $E_F$ is a Fregean thought representing the evidence on which she updates. But again, the attitude expressed by $p_t(P_F \mid E_F, n)$, though it’s expressed here as a future-projected credence in a Fregean thought conditional on another Fregean thought, can equally well be understood as a credence in the centered proposition *P* conditional on supposing that the centered proposition *E* is true and that *n* has passed. And as I suggested above, supposing that *n* has passed can here be understood as supposing, for every proposition in one’s total body of evidence, that its *n*-shifted counterpart is true instead. To model this sort of suppositional belief, we need to generalize our notion of conditional probability so that it allows for suppositional *subtraction* of propositions from the set that makes up an agent’s total evidence as well as for suppositional *addition* of propositions to that set.^16^ So let us stipulate that $p_t(\cdot \mid +\Gamma , -\Delta )$ represents the agent’s state conditional on suppositionally adding the propositions in $\Gamma$ to and subtracting the propositions in $\Delta$ from her total evidence.^17^ Then we can model our agent’s suppositional credence in *P* as follows: $p_t(P\mid +\{E\} \cup C^n, -C)$, where *C* is the agent’s total evidence at *t* and $C^n$ is the set that results from replacing every proposition in *C* with its *n*-shifted counterpart. A few substitutions, then, give us the following:

Self-Certainty Constraint In cases in which a self-certain agent’s dispositions to respond rationally to the passage of time are encoded in her future-projected credences, if she gains some evidence between *t* and $t+n$ that can be modeled at $t+n$ by *E*, then, for any *P*, it should be the case that $p_{t+n}(P) = p_t(P \mid +\{E\} \cup C^n, -C)$.

That is, her attitudes toward centered propositions at $t+n$, just after updating, should match the conditional-on-*E*-and-*n*-having-passed attitudes she has toward those propositions at *t*.^18^

The lesson here, then, can be stated as follows: any generalized Bayesian approach to belief update, if it’s to provide a reasonable treatment of the dynamics of self-location, must deliver the verdicts mandated by the Self-Certainty Constraint. With this lesson in hand, we’re ready to return to the question of whether calibrationism is incompatible with Bayesianism.

## Calibrationism and Bayesianism

Let us, then, reexamine Aisha’s case, keeping in mind now that we’re working under the simplifying assumption that Aisha is a self-certain agent. To recap: $p_S$ models Aisha’s state at 8 pm on Sunday; $p_M$ models her state at 10 am on Monday; *H* is the proposition that, on Monday, she has enough fuel to reach the farther airstrip; *D* is the higher-order evidence that she’s hypoxic at 10 am on Monday; and *E* is the rest of the evidence she gains between 8 pm on Sunday and 10 am on Monday. (As we’ve seen, in cases in which an agent is always certain who she is and where she is in time, it makes no difference from the agent’s perspective whether the propositions under discussion are self-locating propositions or their non–self-locating analogs. So, since Aisha is self-certain, we can assume without loss of generality that *H*, *E*, and *D* aren’t self-locating.)

On the assumption that Aisha is disposed to respond rationally to the passage of time and that these dispositions are encoded in her future-projected credences, the Self-Certainty Constraint entails that $p_M(H)$ should be equal to $p_S(H \mid +\{E \wedge D\} \cup C^{14:00:00}_S, -C_S)$, where $C_S$ is Aisha’s total evidence at 8 pm on Sunday. That is, the constraint entails that Aisha’s unconditional credence at 10 am on Monday in the proposition that she has enough fuel to reach the farther airstrip should match the credence she has in that proposition at 8 pm on Sunday conditional on supposing that she has gained $E \wedge D$ and that fourteen hours have passed. So any reasonable Bayesian approach to belief update that’s general enough to to handle self-locating beliefs will deliver this verdict.

This tells us that a reasonable generalized Bayesian approach is *not* incompatible with the thesis that she should calibrate in the face of *D*: as long as $p_S(H \mid +\{E \wedge D\} \cup C^{14:00:00}_S, -C_S)$ should be less than $p_S(H \mid +\{E\} \cup C^{14:00:00}_S, -C_S)$—i.e., as long as, at 8 pm on Sunday, Aisha should take the proposition that she’s hypoxic at 10 am on Monday to be relevant, on the supposition that fourteen hours have passed, to how likely it is that the calculations she’s performing are correct—any reasonable conditionalization principle will deliver the verdict that she should indeed calibrate. But given Aisha’s self-certainty, she can be certain, on supposing that fourteen hours have passed, that it’s now 10 am on Monday, in which case *D* entails that she’s hypoxic *now*. And the proposition that she’s hypoxic now entails in turn that the calculations she’s performing now are hypoxia-affected. So, insofar as it’s plausible that the information that her calculations are hypoxia-affected is relevant to how confident she should be in those calculations, it’s plausible that $p_S(H \mid +\{E \wedge D\} \cup C^{14:00:00}_S, -C_S)$ should be less than $p_S(H \mid +\{E\} \cup C^{14:00:00}_S, -C_S)$. And it’s *exceedingly* plausible that this information is relevant—indeed, the plausibility of this judgment was what led us to calibrationism in the first place.

It appears, then, that, by generalizing of the notion of conditional probability so as to allow for the modeling of agents’ ability to suppositionally project themselves into the future, we’ve made it quite simple to show that, plausibly, any reasonable Bayesian approach to belief update is compatible with calibrationism. But we should be cautious: despite the plausibility of the claim that $p_S(H \mid +\{E \wedge D\} \cup C^{14:00:00}_S, -C_S)$ should be less than $p_S(H \mid +\{E\} \cup C^{14:00:00}_S, -C_S)$, there remains the question of whether, if $p_S(H \mid +\{E \wedge D\} \cup C^{14:00:00}_S, -C_S)$ meets this constraint, matching $p_M(H)$ to this suppositional attitude is the procedure that maximizes Aisha’s expected accuracy. This is the question to which we now turn.

## Calculating Expected Accuracy

If we’re to answer this question, we must first ensure that we have a complete understanding of how the expected accuracy of an update procedure of this sort is to be calculated. Let’s first consider the standard approach, the one relied on by Greaves and Wallace, on which the expected accuracy, from the point of view of a prior credence function *p*, of a procedure *U* for updating one’s beliefs on undergoing a particular learning experience—in particular, an experience in which the agent knows she’ll gain exactly one new evidence proposition from a specified set $\Gamma$—is calculated as follows:

$$\begin{aligned} EA^{p}(U) = \sum _{X_i \in \Gamma } \sum _{w \in X_i} p(\{w\}) \times A(w, U(X_i)) \end{aligned}$$

where $A(w, U(X_i))$ is the accuracy score, in world *w*, of the credence function that is the outcome of updating on new evidence $X_i$ via procedure *U*. The idea is that, for each world that isn’t entirely ruled out by the prior credence function, there’s one evidence proposition that’s the proposition the agent will gain if that world is actual, and so we can understand the accuracy score of *U*, in a given world, as the accuracy score, in that world, of the probability function that is the outcome of updating on the evidence proposition gained in that world. So, in order to calculate the *expected* accuracy of *U*, we need only weight each of these accuracy scores using the prior credence that the world in question is actual and then sum these weighted scores.

This formula is sensible if the propositions over which the relevant credence functions are defined are understood as sets of possible worlds. But the credence functions we’re interested in are defined instead over *centered* propositions, sets of *centered* worlds. And when centered propositions are in play, this formula leads quickly to absurdity.

The problem arises from the fact that learning experiences take time, and so, in general, the evidence an agent gains on undergoing some learning experience is going to include evidence that time has passed.^19^ Suppose, for instance, that the agent is self-certain and that the learning experience under consideration is the experience she’s going to undergo over some specified amount of time *n*. Then the evidence she gains is going to include evidence that *n* has passed. So, if *t* is the time just before she undergoes the experience and $p_t$ is her credence function at *t*—i.e., her prior credence function—then for any $X_i \in \Gamma$, $X_i$ is going to include evidence that the time is now $t+n$ rather than *t*. Every world in $X_i$, then, is going to be temporally centered on $t+n$. But that means that, for any $w \in X_i$, $p_t(\{w\}) = 0$, since the agent is certain at *t* that the current time is *not* $t+n$. And if that’s right, then, absurdly, $EA^{p_t}(U) = 0$, for any *U*—the expected accuracy of any update procedure is 0.

The explanation for this absurd result is that $p_t(\{w\})$ just isn’t the right prior credence by which to weight the accuracy score $A(w, U(X_i))$: that score, after all, is the accuracy score, in *w*, of the credence function $U(X_i)$, and that credence function is the credence function the agent will have at the *end* of the learning experience—in this case, at $t+n$—if she updates on $X_i$ via *U*. So what determines how this accuracy score contributes to the expected accuracy of *U* is the probability, from the agent’s perspective at *t*, that *w* will be actual at $t+n$, not the probability that *w* is actual at *t*. Or, more generally: in order to calculate the expected accuracy of *U*, we should weight $A(w, U(X_i))$ using the agent’s prior credence that *w* will be actual at the end of the learning experience, not her prior credence that *w* is actual *now*.

This, incidentally, gives us the resources to explain exactly what’s wrong with Schoenfield’s argument that conditionalization fails to maximize expected accuracy when centered propositions are in play. As is clear from her discussion (see her 2018, app. 1), what matters for whether conditionalization maximizes expected accuracy, when centered propositions are *not* in play, is whether the following equivalence holds:

$$\begin{aligned} \sum _{L(X_i) \in L(\Gamma )} \sum _{w \in L(X_i)} p(\{w\}) \times A(w, U(X_i)) = \sum _{X_i \in \Gamma } \sum _{w \in X_i} p(\{w\}) \times A(w, U(X_i)) \end{aligned}$$

where $L(X_i)$ is the proposition that the evidence the agent gains, on undergoing the learning experience, is $X_i$, and where $L(\Gamma )$ is the set of all such propositions. Why? Because, in general, the expected accuracy of *U* is, according to Schoenfield, given by the quantity on the left, not the quantity on the right. And this is what leads to the result that conditionalization maximizes expected accuracy only on the assumption of Evidential Completeness: the equivalence here is guaranteed to hold when Evidential Completeness is satisfied—i.e., when the agent is certain, *before* undergoing the learning experience, that $X_i \leftrightarrow L(X_i)$, for any $X_i \in \Gamma$. But as I’ve just pointed out, the way to calculate the expected accuracy of *U*, when centered propositions *are* in play, is to weight each accuracy score $A(w, U(X_i))$ not by $p(\{w\})$ but by the agent’s prior credence that *w* will be actual at the *end* of the learning experience. So what matters is not whether the above equivalence holds but whether, if $p(\{w\})$ were replaced on both sides by this prior credence, the *resulting* equivalence would hold. And that equivalence is guaranteed to hold not when Evidential Completeness is satisfied but when the agent is certain, for any $X_i \in \Gamma$, that $X_i \leftrightarrow L(X_i)$ will be true at the *end* of the learning experience—i.e., when she takes that evidence on board. That is, it’s guaranteed to hold when the she’s certain that the truth values of evidence propositions are transparent. Schoenfield’s argument, then, since it depends on a false presupposition about how expected accuracy is to be calculated, can give us no reason to doubt that, when centered propositions are in play, conditionalization is the procedure that maximizes expected accuracy.

Moving on: in order to determine how expected accuracy *is* to be calculated when centered propositions are in play, we need to determine how to represent the agent’s prior credence that a given world *w* will be actual at the end of the learning experience. And in the case of a self-certain agent who knows how long the learning experience will take, representing this credence is simple: if such an agent is considering, at *t*, the learning experience she’s going to undergo between *t* and $t+n$, her prior credence that *w* will be actual at the end of the learning experience is just given by $p_t(\{w\}^{-n})$—i.e., her credence, at *t*, in the minus-*n*-shifted counterpart of $\{w\}$ (or, in other words, in the proposition that the centered world that’s actual *now* is the world that’s identical to *w* except that its temporal center has been moved backward by *n*). The expected accuracy of a given update procedure *U*, then, can be calculated as follows:

$$\begin{aligned} EA^{p_t}(U) = \sum _{X_i \in \Gamma } \sum _{w \in X_i} p_t(\{w\}^{-n}) \times A(w, U(X_i)) \end{aligned}$$

This is the correct formula for calculating the expected accuracy of an update procedure when centered propositions are in play, at least in cases in which a self-certain agent knows in advance how long her learning experience will take.

## Maximizing Expected Accuracy

Here we have a bit of a problem. Consider that, given this formula, we can demonstrate, by adapting the reasoning in Greaves and Wallace’s proof, that the procedure that maximizes $EA^{p_t}(U)$ is the procedure *U* that, given any evidence proposition $X_i \in \Gamma$, returns a credence function $p_{t+n}$ such that

$$\begin{aligned} p_{t+n}(P) = p_t(P^{-n} \mid X_i^{-n}) \end{aligned}$$

for any proposition *P*.^20^ Applied to Aisha’s case, this tells us that if she, at 8 pm on Sunday, is considering the learning experience she’s going to undergo between 8 pm on Sunday and 10 am on Monday, the procedure that maximizes $EA^{p_S}(U)$ is the procedure *U* that, given any $X_i \in \Gamma$, returns a credence function $p_M$ such that

$$\begin{aligned} p_M(P) = p_S(P^{-14:00:00} \mid X_i^{-14:00:00}) \end{aligned}$$

for any proposition *P*. (We’ll call this procedure *shifted conditionalization*.) But if that’s right, then, insofar as Aisha should maximize $EA^{p_S}(U)$, the claim that she should update in the way required by the Self-Certainty Constraint entails that she should set her conditional-on-fourteen-hours-having-passed credences in such a way that, for any $X_i \in \Gamma$,

$$\begin{aligned} p_S(P \mid +\{X_i\}, -C_S) = p_S(P^{-14:00:00} \mid X_i^{-14:00:00}) \end{aligned}$$

for any *P*. And from this it follows that, insofar as Aisha should maximize $EA^{p_S}(U)$, our conclusion in Sect. 4 was incorrect. That is, it follows that $p_S(H \mid +\{E \wedge D\} \cup C^{14:00:00}_S, -C_S)$, insofar as Aisha should indeed set $p_M(H)$ equal to that suppositional credence, should *not* be less than $p_S(H \mid +\{E\} \cup C^{14:00:00}_S, -C_S)$. And if that’s right, the thesis that she should update in the way required by the Self-Certainty Constraint *is* incompatible with the thesis that she should calibrate.

To see why, consider that the evidence proposition $X_i$ that Aisha gains, on undergoing her learning experience, can be thought of as a conjunction whose conjuncts are $\langle C^{14:00:00}_S\rangle$ (where this is itself a conjunction whose conjuncts are the members of $C^{14:00:00}_S$) along with whatever other evidence propositions she gains. But in that case, it turns out that, when $X_i$ is shifted *backward* by fourteen hours, the members of $C^{14:00:00}_S$ just turn into the members of $C_S$, which are *already* in her total evidence. Furthermore, if the other evidence propositions she gains are not self-locating, shifting $X_i$ backward by fourteen hours isn’t going to change those evidence propositions at all. So, since *E* and *D* are (we’re supposing) not self-locating, it turns out that, from Aisha’s perspective at 8 pm on Sunday, supposing $X_i^{-14:00:00}$ just amounts to supposing $E \wedge D$ if $X_i = E \wedge D \wedge \langle C^{14:00:00}_S\rangle$, and supposing $X_i^{-14:00:00}$ just amounts to supposing *E* if $X_i = E \wedge \langle C^{14:00:00}_S\rangle$. Finally, since *H* is also (we’re supposing) not self-locating, shifting *it* backward by fourteen hours doesn’t have any effect either: $H = H^{-14:00:00}$. The following, then, are instances of the above equivalence:

$$\begin{aligned}&p_S(H \mid +\{E \wedge D\} \cup C^{14:00:00}_S, -C_S) = p_S(H \mid E \wedge D) \\&p_S(H \mid +\{E\} \cup C^{14:00:00}_S, -C_S) = p_S(H \mid E) \end{aligned}$$

But $p_S(H \mid E \wedge D)$ should be equal to $p_S(H \mid E)$, for reasons we’ve already discussed: the supposition that Aisha will be hypoxic at 10 am on Monday should make no difference to her confidence in her (non–future-projected) calculations on Sunday, when she knows she’s not hypoxic. It follows, then, that $p_S(H \mid +\{E \wedge D\} \cup C^{14:00:00}_S, -C_S) = p_S(H \mid +\{E\} \cup C^{14:00:00}_S, -C_S)$, in which case updating in the way required by the Self-Certainty Constraint involves remaining steadfast, not calibrating.

If we’re to show that calibrating is the procedure that maximizes expected accuracy, we need to explain why this result, despite appearances, doesn’t just immediately entail that Aisha, in order to maximize expected accuracy, should remain steadfast in the face of *D*. And we can indeed explain this, though doing so will require examining one of the assumptions underlying Greaves and Wallace’s way of characterizing update procedures.

Note that, although Greaves and Wallace characterize the update procedures over which expected accuracy is defined as functions from evidence propositions to credal states, they acknowledge that a more general characterization is available: we might instead take an update procedure to be a function from *world states*—in our case, centered worlds—to credal states, so that for an agent to conform to a given procedure *U* is just for her to enter into the credal state *U*(*w*) if *w* is actual. Their result, though, requires that they adopt the more restricted characterization: on the more general characterization, the procedure that maximizes expected accuracy is not conditionalization but the *truth rule*, the function that, for any *w*, returns a credence function *p* such that $p(P) = 1$ if *P* is true in *w* and $p(P) = 0$ otherwise. The justification they offer for adopting the more restricted characterization is that, if a function *U* from world states to credal states isn’t representable as a function from evidence propositions—i.e., if, for some $w_1$ and $w_2$, $U(w_1) \ne U(w_2)$ despite the agent gaining the same evidence in both worlds—then that *U* isn’t an *available* update procedure, since conforming to it “would require the agent to respond to information that he does not have” (2006, p. 612); this is why the truth rule, for instance, doesn’t count as available. Their argument, then, is to be understood as an argument that conditionalization is the *available* procedure that maximizes expected accuracy.

The thought here seems to be that no agent, not even an ideal one, can expect herself to conform to a procedure that requires her to have different beliefs in $w_1$ than she does in $w_2$ despite the fact that she gains exactly the same evidence in both worlds, and if a procedure is such that not even an ideal agent can expect herself to conform to it, it isn’t a procedure that’s available for use in any genuine sense.^21^ But if this is right, then there’s a gap in Greaves and Wallace’s argument: they simply assume that any procedure that *is* representable as a function from evidence propositions to credal states *is* available, and it’s not obvious that this assumption is true. Indeed, unless it’s somehow the case that every ideal agent is certain a priori of her own immunity to hypoxia, reason-distorting drugs, and all other sources of cognitive impairment, the assumption is *not* true—insofar as the agent takes it to be possible that she’s vulnerable to some cognitive impairment or other, there are going to be procedures to which she can’t expect herself to conform despite the fact that they’re representable as functions from evidence propositions to credal states.^22^

Aisha herself is a case in point. Even if she’s in fact a perfect reasoner, she can’t, unless she’s certain a priori that she’s immune to hypoxia, expect, at 8 pm on Sunday, that she’ll conform to an update procedure requiring that, on gaining $E \wedge D \wedge \langle C^{14:00:00}_S \rangle$, she set her credence in *H* equal to $p_S(H \mid E \wedge D)$. After all, on Sunday, she expects, on supposing $E \wedge D$, that she’ll be hypoxic at 10 am on Monday and so will be unable to reliably complete the calculations underlying her current conditional credence $p_S(H \mid E \wedge D)$. Moreover, she expects that, at 10 am on Monday, she won’t have any direct access to this earlier conditional credence, since, by stipulation, either she doesn’t explicitly do any calculations on Sunday or, if she does, hypoxia distorts one’s memories in such a way that her later memories of those calculations aren’t to be trusted. So there just is no mechanism by which she can form new beliefs such that she can expect that $p_M(H)$ will be equal to $p_S(H \mid E \wedge D)$. And if that’s right, then any update procedure, if it requires these credences to be equal, is thereby not an available procedure.

It turns out, then, that shifted conditionalization isn’t available. And if it’s not an available procedure at all, it certainly isn’t the available procedure that maximizes expected accuracy. This is what allows us to avoid the conclusion that Aisha, in order to maximize expected accuracy, should remain steadfast in the face of *D*.

*Is* there an available update procedure such that, if Aisha conforms to it, she’ll remain steadfast? Yes: the update procedure to which she’ll conform simply by doing her calculations on Monday and setting her credence in *H* based on the result of those calculations, without taking *D* into account at all. But *this* procedure doesn’t maximize expected accuracy: we can demonstrate that, if Aisha, on gaining $E \wedge D \wedge \langle C^{14:00:00}_S \rangle$, sets her credence in *H* via this steadfasting procedure, that credence will be less expectedly accurate than a credence arrived at by calibrating.^23^

Note, to begin, that, in order to represent this available steadfasting procedure formally, we need to generalize Greaves and Wallace’s notion of an update procedure in a new way: we need to allow procedures that aren’t deterministic, since, if Aisha expects that she’ll conform to this procedure, she expects that her belief will be based on the verdict returned by calculations that are subject to random errors and so doesn’t expect that a given body of evidence will always result in the same belief. So we can’t represent this steadfasting procedure as a function from evidence propositions to credal states, but we can represent it as a *probability distribution* over functions from evidence propositions to credal states.

To say more about what probability distribution it is, we need to describe the case in a bit more detail. Let’s suppose, then, that $p_S(H) = 0.5$ and that, for any $X_i \in \Gamma$, $p_S(H \mid X_i^{-14:00:00}) = x$ if Aisha’s (non–hypoxia-affected) calculations based on $X_i$ return the verdict that *H* and $p_S(H \mid X_i^{-14:00:00}) = 1- x$ if those calculations return the verdict that $\lnot H$, where $x \gg 0.5$. Then, insofar as Aisha’s update procedure involves setting her credence in *H* based on the verdict returned by her calculations, without taking *D* into account, she’ll respond to gaining $E \wedge D \wedge \langle C^{14:00:00}_S \rangle$ by setting $p_M(H)$ equal to *x* if her (hypoxia-affected) calculations return the verdict that *H* and equal to $1-x$ if these calculations return the verdict that $\lnot H$. Now, to say that hypoxia-affected calculations will return the correct verdict only half the time is just to say that they will return a verdict at random: *H* half the time and $\lnot H$ half the time. So we can represent the steadfasting procedure here as a probability distribution such that the probability of a credal state that assigns *x* to *H* is 0.5 and the probability of a credal state that assigns $1-x$ to *H* is 0.5.

So: what is the expected accuracy, from Aisha’s perspective at 8 pm on Sunday, of using this procedure to update her credence in *H* on gaining $E \wedge D \wedge \langle C^{14:00:00}_S \rangle$? To answer this question, we need a way of scoring the accuracy, in a world, of an individual credence rather than a full credence function. Let’s say, then, that *A*(*P*, *x*) is the accuracy score, in a world where *P* is true, of a credence of *x* in *P*. (Notice that, insofar as accuracy is understood as any sort of function of a credence’s distance from the maximally accurate credence—i.e, a credence of 0 in a falsehood or 1 in a truth—it follows that $A(P, 1 - x)$ is the accuracy score, in a world where *P* is *false*, of a credence of *x* in *P*. We assume here that accuracy is indeed a function of a credence’s distance from the maximally accurate credence.) We also need a way of calculating the *expected* accuracy, in a world *w*, of the credence in *P* that is the outcome of a probabilistic procedure. And this we can calculate straightforwardly, just by taking the accuracy score, in *w*, of the credence in *P* assigned by each possible outcome of the procedure, weighting that score using the probability of that outcome, and summing these weighted scores. Or, more formally:

$$\begin{aligned} EA(w, P, \Pi ) = {\left\{ \begin{array}{ll} \sum _{p \in D(\Pi )} \Pi (p) \times A(P, p(P)) &{} \text {if } w \in P \\ \sum _{p \in D(\Pi )} \Pi (p) \times A(P, 1-p(P)) &{} \text {if } w \notin P \end{array}\right. } \end{aligned}$$

where $\Pi$ is a probability distribution over credal states and where $D(\Pi )$ is the domain of $\Pi$. Finally, we need a way of calculating the expected accuracy, on the supposition that one will gain some evidence *X* between *t* and $t+n$, of the credence in *P* that is the outcome of updating on *X* via a probabilistic procedure. We can calculate this by generalizing our formula for $EA^{p_t}(U)$ to handle probabilistic update procedures and then applying this generalized formula to a case in which the agent is supposing that she’ll gain some particular evidence *X*. If we do so, we get the following:

$$\begin{aligned} EA^{p_t}(X, P, \Pi ) = \sum _{w \in X} p_t(\{w\}^{-n} \mid X^{-n}) \times EA(w, P, \Pi ) \end{aligned}$$

where $\Pi$ is a probability distribution over credal states such that $\Pi (p)$ is the probability that the agent, on gaining *X*, will enter into the credal state *p* .

With these resources in hand, we can calculate the expected accuracy, from Aisha’s perspective at 8 pm on Sunday, of using the available steadfasting procedure to update her credence in *H* on gaining $E \wedge D \wedge \langle C^{14:00:00}_S \rangle$, as follows:

$$\begin{aligned} EA^{p_S}(E \wedge D \wedge \langle C^{14:00:00}_S \rangle , H, \Pi )= & {} \sum _{w \in E \wedge D \wedge \langle C^{14:00:00}_S \rangle } p_S(\{w\}^{-14:00:00} \mid E \wedge D) \\&\times EA(w, H, \Pi ) \end{aligned}$$

So, since the worlds in $E \wedge D \wedge \langle C^{14:00:00}_S \rangle$ can be divided into those worlds that are in *H* and those that aren’t, we have:

$$\begin{aligned} EA^{p_S}(E \wedge D \wedge \langle C^{14:00:00}_S \rangle , H, \Pi )=\ & {} [p_S(H \mid E \wedge D) \times \sum _{p \in D(\Pi )} \Pi (p) \times A(H, p(H))] \\&+[p_S(\lnot H \mid E \wedge D) \times \sum _{p \in D(\Pi )} \Pi (p) \times A(H, 1-p(H))] \end{aligned}$$

Now, by stipulation, Aisha’s calculations were performed correctly despite her hypoxia, and so we know that non–hypoxia-affected calculations would return the verdict that *H* is true. So we know that $p_S(H \mid E \wedge D) = x$. Furthermore, we know that, regardless of whether *H* is actually true, the result of the available steadfasting procedure will be that Aisha’s credence in *H* will be *x* half the time and $1-x$ half the time. By some substitutions, then, we have:

$$\begin{aligned} EA^{p_S}(E \wedge D \wedge \langle C^{14:00:00}_S \rangle , H, \Pi )=\ & {} x \times [0.5 \times A(H, x) + 0.5 \times A(H, 1-x)] \\&+(1-x) \times [0.5 \times A(H, 1-x) + 0.5 \\&\times A(H, 1-(1-x))] \end{aligned}$$

So, simplifying, we have:

$$\begin{aligned} EA^{p_S}(E \wedge D \wedge \langle C^{14:00:00}_S \rangle , H, \Pi ) = 0.5 \times A(H, x) + 0.5 \times A(H, 1-x) \end{aligned}$$

So the expected accuracy, from the perspective of $p_S$, of Aisha’s using the available steadfasting procedure to update her credence in *H* when she gains $E \wedge D \wedge \langle C^{14:00:00}_S \rangle$ is given by this formula.

Note also that the expected accuracy, from the perspective of $p_S$, of a fourteen-hour-later credence of *x* in *H* is just given by a weighted sum in which the accuracy score of that credence in a given world is weighted by Aisha’s confidence, at 8 *pm* on Sunday, that that world will be actual in fourteen hours. Or, more simply, since the accuracy score of a credence in *H* in a world is just a function of whether *H* is true in that world:

$$\begin{aligned} EA^{p_S}(x, H) = p_S(H) \times A(H, x) + p_S(\lnot H) \times A(H, 1 - x) \end{aligned}$$

And since $p_S(H) = p_S(\lnot H) = 0.5$, we have:

$$\begin{aligned} EA^{p_S}(x, H) = 0.5 \times A(H, x) + 0.5 \times A(H, 1 - x) \end{aligned}$$

So, since Aisha assigns accuracy scores in a strictly proper way, we know that $EA^{p_S}(x, H)$ is maximized when $x = 0.5$.

But notice: our formula for $EA^{p_S}(E \wedge D \wedge \langle C^{14:00:00}_S \rangle , H, \Pi )$ and our formula for $EA^{p_S}(x, H)$ are the *same*, and so, since the latter is maximized when $x = 0.5$, we know that the former is maximized when $x = 0.5$ as well. So the available steadfasting procedure, on which $x \gg 0.5$, doesn’t maximize expected accuracy: instead, Aisha will maximize expected accuracy if $x=0.5$. That is, she’ll maximize accuracy if, on gaining $E \wedge D \wedge \langle C^{14:00:00}_S \rangle$, she sets her $p_M(H)$ equal to 0.5 regardless of what verdict is returned by her calculation. Or, in other words, she’ll maximize expected accuracy if she calibrates. So it appears that our conclusion in Sect. 4 was correct after all.

## The Eternal Relevance of Mutation: Concluding Remarks

We’ve arrived, then, at the main results of this paper. The thesis that agents should update their beliefs via the available procedure that maximizes expected accuracy does indeed entail that agents should calibrate in the face of higher-order evidence. Furthermore, accepting this fact doesn’t require abandoning a Bayesian approach to belief update: any Bayesian approach that’s generalized in such a way as to provide a reasonable treatment of the dynamics of self-location will thereby be compatible with calibrationism.

It turns out, though, that these results have an implication that’s somewhat surprising, and so we’ll conclude with a mention of this implication and its bearing on another debate about the dynamics of self-location.

Bradley (2011) distinguishes between two kinds of evidence acquisition: *discovery*, in which an agent gains new information about something about which she was previously uncertain, and *mutation*, in which an agent changes her state of information by tracking changes in the truth values of propositions over time. Evidence propositions that aren’t temporally self-locating—i.e., evidence propositions that are *eternal*—don’t change in truth value over time and so can only be acquired by discovery. But temporally self-locating evidence propositions, such as the proposition that the clock now reads 3 pm, can be acquired in either way: an agent might gain this evidence by glancing at the clock, having completely lost track of what time it is, or she might gain it by staring at the clock as it ticks over from 2:59 pm.

One important difference between discovery and mutation, Bradley says, is that, unlike discovery, mutation is never relevant to one’s eternal beliefs. That is, when one gains new evidence by mutation, it’s never rational, according to Bradley, to respond to that evidence by changing one’s credences in eternal propositions. And this entails that, in the famous Sleeping Beauty problem, Beauty’s credence that the coin landed heads, on waking, should be 0.5, since (i) it’s uncontroversial that her credence should be 0.5 at the start of the experiment, (ii) the only new evidence she gains on waking is gained by mutation, and (iii) the proposition that the coin landed heads is eternal.

Bradley’s argument for the claim that mutation is never relevant to eternal beliefs is that only if this claim is true can we can maintain that conditionalization as standardly formulated is the only update procedure that governs eternal beliefs, and “giving up a principle as defensible and reasonable as conditionalization is a heavy cost” (2011, p. 408). This appears to be what motivates his halfer position about the Sleeping Beauty problem, and it also appears to be what motivates him to reject as insufficiently convincing a variety of arguments given by thirders the conclusions of which entail that mutation *is* eternally relevant.^24^ It turns out, though, that the results at which we’ve arrived here make available a novel argument for the eternal relevance of mutation.

Consider a slightly altered version of Aisha’s case, one in which, at 8 pm on Sunday, she already knows everything that’s going to happen to her over the next fourteen hours. That is, the experiences she has over those fourteen hours are the same as in the original case, but they don’t give her any new evidence other than evidence about where she is in time, since she knows in advance that she’s going to have those experiences. This is a paradigm case of mutation: between 8 pm on Sunday and 10 am on Monday, she doesn’t learn anything about which she was previously uncertain but simply tracks changes in the truth values of certain propositions. So it follows, on Bradley’s view, that her credences in eternal propositions shouldn’t change during that time. In particular, $p_M(H)$ should be equal to $p_S(H)$.

This is precisely the result delivered by shifted conditionalization.^25^ But for reasons we’ve already seen, shifted conditionalization isn’t an available procedure, nor is any other procedure that delivers this result—Aisha, on Sunday, expects that she’ll be hypoxic and so will be unable to reliably complete the calculations underlying her earlier credence in *H*, and she also expects that she won’t be able to access this earlier credence directly, since she either doesn’t explicitly perform any calculations on Sunday or is unable on Monday to trust the memories she seems to have of the results of these earlier calculations. In short, the argument of Sect. 6 works in this altered version of Aisha’s case in just the same way as it does in the original case: the considerations in favor of calibrating aren’t in any way sensitive to whether Aisha knows $E \wedge D$ in advance.

This altered version of Aisha’s case, then, is a counterexample to the thesis that mutation is never relevant to eternal beliefs.^26^ So, insofar as attachment to Sleeping-Beauty halferism is motivated by a desire to maintain that thesis, this attachment is simply unwarranted.

## Footnotes

1 For early discussions of this case, see Elga (2008) and Christensen (2010b). I’ve chosen to focus on this case so that I can more easily emphasize the points of contact between my discussion and that of Schoenfield (2018), who also focuses on a version of it.

2 The phenomenon here was, as far as I know, first pointed out by Christensen (2010a, pp. 199–200).

3 Versions of this view are defended by, e.g., Lasonen-Aarnio (2014), Titelbaum (2015), Weatherson (2019, chap. 11), and Whiting (2019).

4 See, e.g., Arntzenius (2003) and Hitchcock (2004).

5 Schoenfield (2015, 2018) introduces a nonstandard picture of the connection between expected accuracy and the rational status of update procedures and suggests that adopting this picture will allow us to “deliver an accuracy based argument for calibrating” (2018, p. 711). But this, I argue in my (2021), is incorrect: Schoenfield’s alternative picture turns out always to agree with the standard picture about what update procedure is rational, and so, whatever other reasons there may be for adopting Schoenfield’s picture, doing so will be of no help in resolving the apparent incompatibility between calibrationism and the accuracy-first program.

6 On Schoenfield’s presentation, there are two conditions that need to be satisfied, which she calls *Partitionality* and *Factivity*. But she proves (in her 2018, app. 1 and also, with additional commentary, in her 2017, sec. 2) that the conjunction of these two conditions is equivalent to Evidential Completeness.

7 The proof appears in Schoenfield (2018, app. 2) and also, with additional commentary, in Schoenfield (2017, sec. 3).

8 If it seems odd that what Greaves and Wallace’s result requires is that Evidential Completeness be satisfied rather than that the agent be sure that the truth values of evidence propositions are transparent, that’s because it *is* odd. The fact that this is what’s required just follows from the way the expected accuracy of an update procedure is defined, but as I explain in Sect. 5 below, this definition, when centered propositions are in play, fails to capture what it’s intended to capture and so stands in need of revision.

9 Even more strikingly: if Aisha does perfectly track time, then if *N* is the centered proposition she’d express by “It’s now Sunday night”, then $p_S(N) = 1$. And if $p_S(N) = 1$, then $p_S(N \mid E \wedge D_c) = 1$, since conditional credences preserve certainty. So, on the view Schoenfield takes to be her target, it should be the case that $p_M(N) = 1$: Aisha, at 10 am on Monday, should be certain that it’s now Sunday night. But this result is obviously absurd. (Incidentally, the view that Aisha should update by conditionalization* has this same problem, though Schoenfield seems not to have noticed this. See Bradley (2020) for some additional worries about conditionalization*.)

10 As Frege puts it: “Everyone is presented to himself in a particular and primitive way, in which he is presented to no-one else” (1918/1956, p. 298).

11 Note that that the view here is distinct from that defended by Perry (1979) and Kaplan (1989), on which to have a self-locating belief is to be related in a particular way to a *de re* proposition. Being related in different ways at different times to the same *de re* proposition need not be a manifestation of an ability to keep track of time. See Bradley (2013) for an attempt to model the kind of belief persistence I’m discussing from within the sort of framework favored by Perry and Kaplan.

12 Incidentally, we won’t be discussing in detail this framework’s treatment of cases in which an agent loses track of time—again, modeling these cases isn’t going to be important for our purposes here. But, very briefly: losing track of time, on this framework, is to be understood as a kind of forgetting.

13 This simple approach is available only because we’re dealing with agents who are always sure where they are in time. As I’ve already suggested, a Fregean approach to modeling the temporally self-locating beliefs of agents who aren’t self-certain would be much more complicated.

14 We’re assuming for simplicity that, in ideal cases, updating one’s beliefs doesn’t take any time.

15 It’s superficially plausible that, since the truth values of Fregean thoughts don’t change over time, projecting oneself into the future should make no difference to one’s attitudes toward those thoughts, in which case $p_t(\cdot \mid E_F, n)$ should be equal to $p_t(\cdot \mid E_F, 0)$. But this is a version of what Titelbaum (2008) calls the *Relevance-Limiting Thesis*—roughly, that if the only new information one gains is self-locating information (i.e., if one’s new information doesn’t rule out any non–self-locating possibilities), then it can never be rational to change beliefs the contents of which aren’t self-locating—and that thesis is controversial. Titelbaum, for instance, argues that it’s false, though he accepts a restricted version of it that applies in cases in which the agent is self-certain (see his 2013, chap. 10 and his 2016). In Sects. 6–7 we’ll see that the phenomenon of higher-order defeat gives us some reason to think that even this restricted version is false.

16 Note that, following, e.g., Price (1986), Hájek (2003), and Titelbaum (2022), I am here taking conditional credence (both in its usual form and in this generalized form) to be mathematically primitive: conditional credences aren’t defined in terms of unconditional credences but instead directly model agents’ suppositional degrees of belief. So there is no mathematical definition to be given of this generalized notion of conditional credence.

17 Titelbaum (2013) introduces a formal framework that allows for the modeling of certainty loss over time, but he opts not to countenance beliefs conditional on certainly false suppositions, on the grounds that adding such a supposition to one’s total evidence results in “a situation partially defined by the truth of a logical contradiction” (2013, p. 103). Generalizing the notion of conditional probability so as to allow for suppositional subtraction gives us a *formal* way around this problem, but there remains the question of whether there’s good psychological sense to be made of an agent’s having beliefs conditional on the removal of propositions from her evidence set. We don’t need to take any stand here about whether there’s sense to be made of this sort of suppositional subtraction in general, but I take it that it’s clear enough that the particular cases under discussion here—cases in which an agent replaces the propositions in her evidence set with shifted counterparts so as to suppositionally project herself into the future—make good psychological sense. See also Chalmers’s (2011, pp. 631–632) defense of the view that, in cases in which a certainly false centered proposition may become true in the future, an agent can have (and can conditionalize on) credences conditional on that centered proposition.

18 If we translate the version of the Relevance-Limiting Thesis mentioned in fn. 15 into the centered-proposition framework, what we get is the thesis that $p_t(P \mid +\{E\} \cup C^n, -C)$ should be equal to $p_t(P^{-n} \mid E^{-n})$, that the agent’s attitudes toward centered propositions on supposing *n* has passed should match her present attitudes toward the minus-*n*-shifted counterparts of those propositions. If this thesis were correct, we could conclude that $p_{t+n}(P)$ should be equal to $p_t(P^{-n} \mid E^{-n})$—i.e., that self-certain agents should conform to a version of the update procedure Schulz (2010) calls *continuous conditionalization* and Schwarz (2012, 2015, 2017) calls *(SC)*, or *shifted conditioning*. (Both Schulz and Schwarz endorse this procedure as a rational requirement in cases of agents that don’t lose track of time. Note also that quite a few of the generalizations of conditionalization that have been proposed for handling temporal self-location—e.g., those proposed by Kim (2009), Briggs (2010), Meacham (2010), Moss (2012), and Spohn (2017)—reduce to this procedure in cases of self-certainty.) But again, there’s reason to think the Relevance-Limiting Thesis is false. I explain in Sect. 6 why setting $p_{t+n}(P)$ equal to $p_t(P^{-n} \mid E^{-n})$ isn’t the update procedure that maximizes expected accuracy.

19 Strictly speaking, there will be temporal evidence propositions lost as well as temporal evidence propositions gained, for reasons discussed above. But there’s no need here to explicitly represent the loss of the old propositions—that loss is implicit in the gaining of the new ones, since the new ones just entail that the old ones are false.

20 Although the required adaptation of Greaves and Wallace’s reasoning is straightforward, tracing that reasoning in detail would require introducing conceptual resources that are beyond the scope of our discussion. But note that Schwarz (2015, sec. 6) argues, by adapting a proof given by Leitgeb and Pettigrew (2010) that’s similar but not identical to the one given by Greaves and Wallace, that the above procedure does maximize expected accuracy. (Recall that, as mentioned in fn. 18, this is the procedure Schwarz calls *shifted conditioning*.)

21 For further discussion, see my (2021).

22 For discussion of why ideal agents plausibly aren’t certain of their immunity to cognitive impairment, see, e.g., Christensen (2007).

23 The reasoning in this section is adapted from an argument given by Schoenfield (2018, sec. 6) though she, again, relies on a nonstandard picture of the connection between expected accuracy and the rational status of update procedures. Part of my reason for adapting this argument is to show that, in fact, no recourse to Schoenfield’s nonstandard picture is necessary. (This is unsurprising given the argument in my (2021), the upshot of which is that Schoenfield’s picture and the standard picture always agree.)

24 In replying to the argument given by Elga (2000), for instance, he says the following: “Keep in mind that Elga is arguing that we should do something radical i.e. substantially revise our best theory of confirmation, so we should require a strong argument to do so” (2011, p. 405).

25 Shifted conditionalization, recall, is such that, if Aisha conforms to it, $p_M(H)$ will be equal to $p_S(H \mid X_i^{-14:00:00})$, where $X_i$ is the evidence she gains. But we’ve seen that, for any evidence proposition she gains just as a matter of tracking the passage of time, shifting that proposition backward by fourteen hours will turn it into a proposition that’s already in her total evidence. To conform to shifted conditionalization, then, is to set $p_M(H)$ equal to $p_S(H \mid N^{-14:00:00})$, where *N* is the evidence she gains *other than* what she gains just as a matter of tracking the passage of time. So, since the case here is one in which she doesn’t gain any other evidence, $p_M(H) = p_S(H)$.

26 Incidentally, it’s also a counterexample to the Relevance-Limiting Thesis discussed in fns. 15 and 18. Indeed, it’s a counterexample that doesn’t require that the agent ever be less than certain about who she is or where she is in time, and so it’s a counterexample even to the restricted version of that thesis endorsed by Titelbaum.
